# Phytotoxic and Antimicrobial Activities of *Teucrium polium* and *Thymus decussatus* Essential Oils Extracted Using Hydrodistillation and Microwave-Assisted Techniques

**DOI:** 10.3390/plants9060716

**Published:** 2020-06-04

**Authors:** Ibrahim Saleh, Ahmed Abd-ElGawad, Abd El-Nasser El Gendy, Abeer Abd El Aty, Tarik Mohamed, Hazem Kassem, Fahd Aldosri, Abdelsamed Elshamy, Mohamed-Elamir F. Hegazy

**Affiliations:** 1 Chemistry of Medicinal Plants Department, National Research Centre, 33 El-Bohouth St., Dokki, Giza 12622, Egypt; brahim82@hotmail.com (I.S.); tarik.nrc83@yahoo.com (T.M.); 2 Plant Production Department, College of Food & Agriculture Sciences, King Saud University, P.O. Box 2460, Riyadh 11451, Saudi Arabia; 3 Department of Botany, Faculty of Science, Mansoura University, Mansoura 35516, Egypt; 4 Medicinal and Aromatic Plants Research Department, National Research Centre, 33 El Bohouth St., Dokki, Giza 12622, Egypt; aggundy_5@yahoo.com; 5 Department of Biology, College of Science, University of Hafr Al Batin, P.O. Box 1803, Hafr Al Batin 39524, Saudi Arabia; abeerab@uhb.edu.sa; 6 Chemistry of Natural & Microbial Products Department, National Research Centre, Dokki, Giza 12622, Egypt; 7 Agricultural Extension and Rural Society Dept., College of Food & Agriculture Sciences, King Saud University, P.O. Box 2460, Riyadh 11451, Saudi Arabia; hskassem@ksu.edu.sa (H.K.); fadosri@ksu.edu.sa (F.A.); 8 Faculty of Pharmaceutical Sciences, Tokushima Bunri University, Yamashiro-cho, Tokushima 770-8514, Japan; elshamynrc@yahoo.com; 9 Chemistry of Natural Compounds Department, National Research Centre, 33 El Bohouth St., Dokki, Giza 12622, Egypt

**Keywords:** Lamiaceae, *Teucrium polium*, *Thymus decussatus*, essential oils, phytotoxicity, antimicrobial

## Abstract

Essential oils (EOs) have been described as promising eco-friendly secondary products of aromatic plants with several biological activities. The present study aimed to characterize the chemical composition and explore phytotoxic and antimicrobial activities of *Teucrium polium* and *Thymus decussatus* EOs extracted using hydrodistillation (HD) and microwave-assisted extraction (MAE) methods. Twenty-seven and twenty-eight compounds were identified from HD and MAE extracted EOs of *T. polium*, respectively. The oxygenated sesquiterpenes (57.68%) were characterized as the main components of the hydrodistilled EO with a prominence of 6-epi-shyobunol (33.00%), while sesquiterpene hydrocarbons (54.48%) were the main components of the MAE method, with a prominence of delta-cadinene (25.13%). Eighteen and nineteen compounds, were characterized in *T. decussatus* EOs extracted using HD and MAE methods, respectively, and oxygenated monoterpenes represented the main components of both EOs with carvacrol (94.40% and 75.91%, respectively) as the main compound. The EOs extracted using the MAE method were slightly more phytotoxic than those extracted using the HD method. The *T. decussatus* EO extracted using the MAE method showed a higher inhibitory effect than *T. polium* by 16-, 32-, and 24-fold, regarding seed germination, shoot, and root growth of lettuce, respectively. Moreover, EOs extracted by HD method showed a similar pattern with 16-, 28-, and 14-fold effects. Both *T. decussatus* EOs exhibited potent inhibitory effect against all tested bacteria with an inhibition zone of 34–39 mm and the lowest minimum inhibitory concentration (MIC) of 0.49, 0.98, and 1.95 μg/mL against *Aspergillus niger*, *Escherichia coli,* and *Staphylococcus aureus*, respectively. However, the EOs of *T. polium* showed weak antibacterial activity and no antifungal effect. Further studies are needed for the characterization of bioactive major compounds, either singular or synergistic, at field scale and to determine their modes of action and safety.

## 1. Introduction

From the first days of ancient civilizations, especially the Egyptian one, medicinal herbs have been used as the main source of medicinal agents. Egypt is one of the countries that is characterized by unique biodiversity. With ecological variations, from deserts to seas (the Mediterranean and Red Seas), the Nile River, Nile delta, oases, depressions, and mountains [1], Egypt is also characterized with verifications of medicinal plants, including 529 medicinal plants, 60 endemic plants, and 13 pharmacopeias [2,3]. The Sinai Peninsula, especially the Saint Katherine protectorate, represents the most promising source of traditional herbs in Egypt. The plant species collected from Sinai are characterized by unique metabolites and potent pharmacological effects [4,5].

Aromatic plants are considered a natural source of essential oils (EOs). EOs are considered as a promising source of complex mixtures of metabolites, especially terpenoids [6,7]. Many biological activities have been investigated for EOs, such as phytotoxic [8,9,10], antimicrobial [11], anti-inflammatory, antipyretic [12], antiulcer [13], and hepatoprotective activities [14]. The Lamiaceae (Labiatae) family is considered an important family, having many aromatic plants. Lamiaceae contains 236 genera that embrace 6900–7200 species [15,16]. The most famous genera of the Lamiaceae family are *Salvia*, *Scutellaria*, *Stachys*, *Plectranthus*, *Hyptis*, *Teucrium*, *Vitex*, *Thymus*, and *Nepeta* [16].

*T. polium* L., one of the most important aromatic plants, is described as an important medicinal plant with several traditional uses, such as treatment of gastrointestinal disorders, inflammations, diabetes, and rheumatologic diseases [17]. In addition, many biological activities were deduced for different extracts of this plant, such as antioxidant, hepatoprotective [17,18], anti-cancer, antimicrobial, antinociceptive, and analgesic activities [19]. The reported chemical characterization confirmed that this plant is auspicious, and has high terpenoid content, especially sesquiterpenes [20,21], iridoid glycoside [21], triterpenes [20], phenyl-ethanoid glycosides, and flavonoids [20,21]. Some chemical profiles and biological investigations of the EOs of *T. polium* were investigated around the world, as in Iran [22], Tunisia [23], and Saudi Arabia [24].

On the other hand, plants belonging to *Thymus* genus are described to have numerous traditional applications for several diseases, such as headache, ulcers, eczema, renal diseases, asthenia, wounds, verrucae, and diabetes [25]. *T. decussatus* Benth. is a very rare plant growing in Saini, Egypt [3]. This plant is very famous for the treatment of nausea [25]. Few reports described *T. decussatus* EO chemical profiles and biological activities, including antimicrobial and cytotoxicity activities [26,27].

Continuing our target for finding eco-friendly natural bioactive compounds [6,7,12,21,28,29,30,31], our study aimed to (1) characterize the chemical profiles of the EOs of two medicinal plants belonging to Family Lamiaceae, *T. polium* and *T. decussatus*, collected from the Saint Katherine Protectorate, Sinai Peninsula, Egypt, (2) evaluate the phytotoxic effects of the extracted EOs on *Lactuca sativa* (lettuce), (2) determine the antimicrobial activities of EOs on different microorganisms, and (4) evaluate the significance of the extraction method on the chemical composition via chemometrics analysis.

## 2. Results and Discussion

### 2.1. Chemical Profiles of EOs

The EOs of *T. polium* were extracted using hydrodistillation (HD) and microwave-assisted extraction (MAE) methods and produced 0.045% and 0.061% (*v/w*) of yellow oils, respectively. The variation of the oil yield might be attributed to the extraction method used [32]. The extracted EOs were analyzed by Gas chromatography–mass spectrometry (GC–MS) and the chemical constituents were identified and described in Table 1. Twenty-seven compounds were identified from the hydro-distilled EO, representing 100% of the total mass, while 28 compounds were identified from MAE extracted EO.

In the case of the hydro-distilled EO sample, oxygenated sesquiterpenes were characterized as the main components, with a concentration of 57.68%, followed by monoterpene hydrocarbons (24.12%) and sesquiterpene hydrocarbons (16.52%), with the absence of non-terpenoid compounds. Oxygenated sesquiterpene, 6-epi-shyobunol (33.00%), was found to be the main compound, followed by *t*-muurolol (12.88%) and germacrene D-4-ol (5.65%). Delta-cadinene (7.04%) and aromadendrene (3.86%) were identified as the major compounds of the sesquiterpene hydrocarbons.

The results showed that sesquiterpene hydrocarbons (54.48%) were found as the main identified compounds of the *T. polium* EO extracted using the MAE method, followed by monoterpene hydrocarbons (23.52%) and oxygenated sesquiterpenes (22.0%). In the case of the MAE method, delta-cadinene (25.13%) was found to be the main compound of the identified sesquiterpene hydrocarbons, alongside *α*-muurolene (7.64%), germacrene-D (7.25%), and *β*-muurolene (5.55%). τ-cadinol (7.61%), alloaromadendrene oxide (6.09%), and *α*-cadinol (5.49%) were identified as the main oxygenated sesquiterpene compounds, while *α*-pinene (11.58%) and *β*-pinene (8.20%) were found as the main constituents of the identified monoterpene hydrocarbons, with a complete absence of oxygenated monoterpenes.

The preponderance of sesquiterpenes in the present study is consistent with other studies reporting the chemical characterization of EOs derived from other ecotypes of *T. polium* growing in Jordan [33], France [34], Algeria [35,36,37], Serbia, the Balkans [38], and Iran [39].

However, other studies showed a different composition of *T. polium* EOs [40,41,42,43,44], and this discrepancy in the composition could be ascribed to the variation in the environmental and climatic conditions [7,9]. This observation revealed the presence of various chemotypes of *T. polium*. Therefore, further study is needed for the discrimination of this species at a molecular level.

It is worth mentioning here that in the Egyptian flora, *T. polium* is mentioned without reference to any subspecies [3,45]. Therefore, from a taxonomic point of view, our results recommend further identification of Egyptian ecospecies of *T. polium*, which could help for more detailed identification at the sublevel.

On the other hand, the EOs extracted from *T. decussatus* by HD and MAE were colorless or faint yellow with a yield of 0.023% and 0.031% (*v*/*w*), respectively. This variation could also be ascribed to the method of extraction used [32]. Significant variations of the chemical compositions and antimicrobial activities were described among the extracted EOs of *Artemisia argyi* by HD, simultaneous distillation, and subcritical extraction techniques [46]. Moreover, the yield and composition of the EOs from *Carum carvi*, *Anethum graveolens*, and *Pimpinella anisum* were varied according to the method of extraction used [47].

In the case of *T. decussatus*, GC–MS analysis of the extracted oil revealed a lower number of identified compounds compared to *T. polium* (Table 1). The results also showed that the type of extraction method used affected the chemical profile of the extracted EO. Extraction using HD showed the presence of eighteen compounds, while the extracted EO with MAE method showed the presence of nineteen compounds. Monoterpenes were the main identified components of both extracted EOs, using HD and MAE methods, with concentrations of 99.19% and 98.53%, respectively. Overall, the identified monoterpenes were categorized into (1) oxygenated monoterpenes (94.53% and 76.93%, respectively) and (2) monoterpene hydrocarbons (4.66% and 21.59%, respectively). Carvacrol (94.40% and 75.91%, respectively) was the main oxygenated monoterpene identified, while *p*-cymene (3.61% and 16.98%, respectively) was the main identified monoterpene hydrocarbon. In the present study, the superiority of monoterpenes in the EOs of *T. decussatus* is in complete agreement with the previously documented studies of the EOs of Egyptian *T. decussatus* [26], as well as Italian *T. pulegioides* [48]. In contrast to our results, De Martino, et al. [48] described that the EO of *T. longicaulis* collected from Italy is rich with sesquiterpenes. These chemical diversities could be attributed to different environmental and genetic variations, for example plant age, seasonality, temperature, moisture, altitude, nutrient availability, and salinity variations [6].

Numerous reports described that the diterpenoids are rare and/or completely absent constituents in EOs derived from plants, with some exceptions [8]. The results of GC–MS of all extracted EOs from the two plants, *T. polium* and *T. decussatus*, deduced this to be true by the complete absence of diterpenoids.

From all the discussed data, the variation of the chemical constituents of the EOs is deduced to the correlation with the variations in plant organ, collection place, and environmental conditions including salinity, temperature, etc., as described in several reports. Additionally, our study supported the previously documented results that deduced the variations of chemical compositions of EOs with the extraction method used.

### 2.2. Principal Component Analysis (PCA) of EO Chemical Compounds

**Figure 1:**
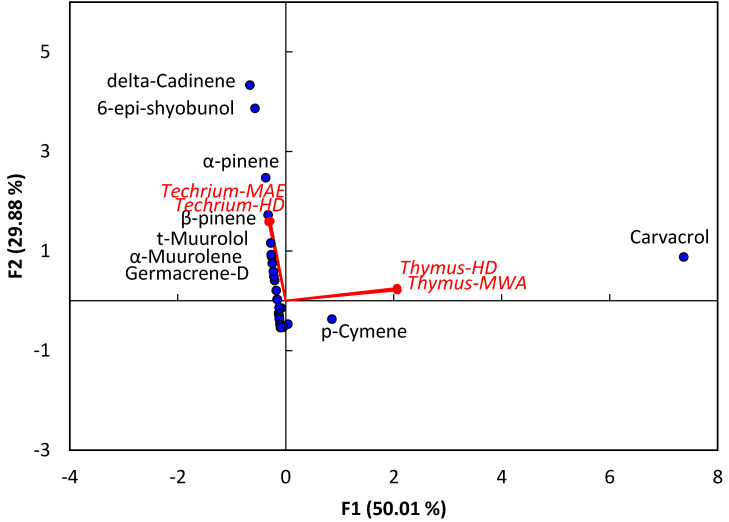
Principal component analysis (PCA) of EO compounds extracted from *Teucrium polium* and *Thymus decussatus* using microwave-assisted extraction (MAE) and hydrodistillation (HD) methods.

The PCA analysis of the EO chemical compositions showed the difference between the EOs of *T. polium* and *T. decussatus* (Figure 1). Moreover, both samples of the extracted EOs (HD and MAE) are closely correlated for both plant species. Regarding the variables (compounds), *T. polium* EOs showed a correlation with *α*-pinene, *β*-pinene, *t*-muurolol, *α*-muurolene, germacrene-D, delta-cadinene, and 6-epi-shyobunol. However, the EOs of *T. decussatus* revealed a correlation with carvacrol and *p*-cymene.

### 2.3. Phytotoxicity Activity

The EOs of both *T. polium* and *T. decussatus*, extracted using the HD and MAE methods, exhibited a significant phytotoxic effect on the tested plant; *L. sativa*, and the inhibition was dose-dependent (Table 2). Compared to *T. polium* EOs, *T. decussatus* EOs showed more a phytotoxic effect at the highest concentration (100 μL L^−1^), as *T. decussatus* EO extracted using HD method inhibited the seed germination, shoot growth, and root growth of lettuce by 86.6%, 87.4%, and 89.9%, respectively, while the EO extracted using the MAE method inhibited lettuce by 77.7%, 85.8%, and 84.6%, respectively.

On the other hand, the EO extracted using HD from the above-ground parts of *T. polium* inhibited the seed germination, shoot growth, and root growth of lettuce by 51.9%, 31.4%, and 54.4%, respectively, at a concentration of 1000 μL L^−1^, while the EO extracted using MAE method reduced the seed germination, shoot growth, and root growth of lettuce by 50.9%, 39.8%, and 42.3%, respectively (Table 2).

**Figure 2:**
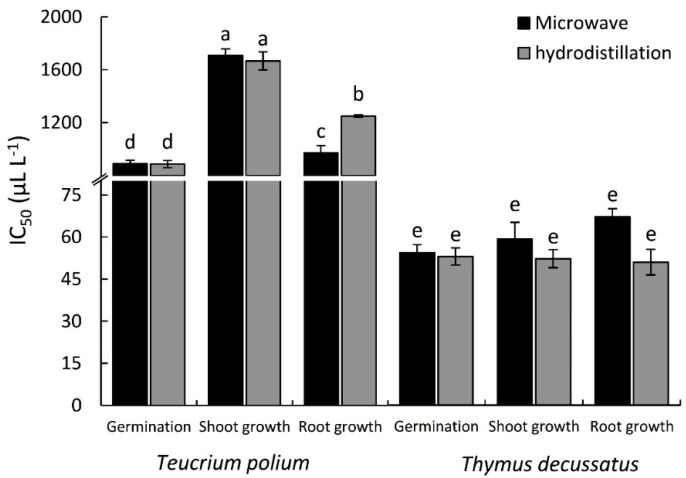
The IC_50_ values of the essential oils extracted from *Teucrium polium* and *Thymus decussatus* using the microwave-assisted extraction (MAE) and hydrodistillation (HD) methods, based on the inhibition of the seed germination and seedling growth of lettuce. Different letters of columns mean values are significantly different at *p* ≤ 0.05.

Based on the IC_50_ values, the EOs extracted by MAE method showed a slightly higher phytotoxic activity than those extracted by HD method (Figure 2). However, the EO extracted using the MAE method from *T. decussatus* showed a higher inhibitory effect than *T. polium* by 16-, 32-, and 24-fold, regarding the seed germination, shoot growth, and root growth of lettuce, respectively. Moreover, the EO extracted by HD showed a similar pattern with a 16-, 28-, and 14-fold change, respectively (Figure 2).

Summing up, the data reflect the high potency of *T. decussatus* EOs, which could be attributed to its content of terpenoid compounds, particularly the monoterpenes, such as the major compounds carvacrol and *p*-cymene. It is worth mentioning that *p*-cymene is represented as the major phytotoxic compound of many plants, such as *Origanum acutidens* [49], *Zataria multiflora* [50], *Eucalyptus citriodora*, *Eucalyptus grandis* [51], *Thymbra spicata* [52], *Thymus eigii* [53], and *Thymus daenensis* [54].

It is important here to mention that oxygenated terpenoid compounds showed more phytotoxic activity than non-oxygenated compounds [6,7,9]. Carvacrol represents 94.4% and 75.9% of the total mass of the EOs extracted by HD and MAE methods from *T. decussatus*, respectively. Thereby, it could play the main role of the phytotoxic activity of both the extracted EOs. Additionally, carvacrol was previously reported to exhibit phytotoxic activity in many plants, such as *Plectranthus amboinicus* [55], *Melissa officinalis*, *Origanum vulgare* [56], *Zataria multiflora* [50], *Eriocephalus africanus* [57], *Origanum acutidens* [49], *Thymbra spicata* [52], *Thymus eigii* [53], *Satureja* [58], and *Thymus daenensis* [54].

The mode of action of carvacrol is attributed to the presence of the free hydroxyl group and proton exchange delocalized system [59]. This mode of action is ascribed to the antimicrobial activity of the carvacrol [60]. By simulation, it could also be responsible for the phytotoxic activity in the present study.

### 2.4. Antimicrobial Activities

The extracted EOs using HD and MAE methods showed variable antimicrobial activities (Table 3 and Table 4). The EOs of *T. polium*, extracted by HD and MAE methods, showed only antibacterial activity against the tested strains (Gram-positive and Gram-negative bacteria). However, the EOs of *T. decussatus* revealed a higher activity, as they showed both antibacterial and antifungal activities. *T. decussatus* EOs extracted using two different extraction methods exhibited a potent inhibitory effect against all bacterial strains, with an inhibition zone of 34–39 mm, and very strong antifungal activity, especially against the filamentous fungi, *Aspergillus niger* and *Fusarium solani*, with an inhibition zone of 40–48 mm.

Based on the data of the MICs, both samples of *Teucrium polium* EOs (HD and MAE) showed MIC values of 62.50 and 125.00 μg/mL against *B. subtilis* and *S. aureus*, respectively, while they showed an MIC of 250.00 μg/mL against Gram-negative bacteria (*E. coli*). On the other hand, the *T. decussatus* EOs revealed MIC values of 3.91, 1.95, and 0.98 μg/mL against *B. subtilis*, *S. aureus*, and *E. coli*, respectively. Additionally, *T. decussatus* EOs showed strong antifungal activity on the tested fungi, with MIC values of 0.49, 3.91, and 7.81 μg/mL against *A. niger*, *C. albicans*, and *F*. *solani*, respectively (Table 4).

Belmekki, et al. [61] reported moderate antimicrobial activity of the EO from Algerian *T. polium* with MIC value of 3 to 5 µL/mL. Meanwhile, another study by Kerbouche, et al. [37], reported a two-fold increase in the antimicrobial activity of the *T. polium* ecospecies growing in Algeria. The reported antimicrobial activity of the EOs of *T. polium* could be attributed to the sesquiterpenes, particularly the oxygenated compounds such as 6-epi-shyobunol, *t*-muurolol, and germacrene D-4-ol.

The strong antimicrobial activity of *T. decussatus* could be attributed to the major compounds of the EO, such as carvacrol and *p*-cymene. Several studies reported the antimicrobial activity of carvacrol and *p*-cymene [59,62,63,64].

## 3. Materials and Methods

### 3.1. Plant Materials

The above-ground parts of *T. polium* and *T. decussatus* were collected from Wadi Jibaal in St Katherine Protectorate (28°32′32″ N 33°57′30.2″ E), south Sinai, Egypt, during the flowering stage. Voucher specimens (SK-105 (*T. polium*) and SK122 (*T. decussatus*)) have been deposited in the herbarium of the National Research Centre, Cairo, Egypt. The collection took place under the permission of the St Katherine Protectorate for scientific purposes. The specimens were authenticated by Dr. Ahmed Abd-ElGawad, Associate Professor of Plant Ecology, Faculty of Science, Mansoura University, Egypt.

### 3.2. EO Extraction, Analysis, and Constituent Identification

The EOs of the aerial parts (300 gm, each) of *T. polium* and *T. decussatus* were extracted separately via the HD and MAE methods, analyzed by GC–MS, and the constituents were identified as described previously by our team [8,32]. In brief, the HD-EOs were extracted via a Clevenger-type apparatus for 3 h. Meanwhile, the extractions of the MAE-EOs were performed using a focused microwave apparatus (CEM Corporation, Matthews, NC, USA), model (MARS 240/50, No. 907511, frequency 2450 MHz) operating at 2450 MHz with a maximum power of 1600 W. A sample of each plant was separately placed in a 5000 mL round-bottomed flask connected to a Clevenger-type apparatus outside of a microwave oven. The extraction was operated using 800 W power for 60 min. The temperature was adjusted to 100 °C. The essential oil was recovered and its volume was determined using a micropipette. The oily layer was separated using diethyl ether and dried with anhydrous sodium sulfate (0.5 g). This extraction was repeated two times and afforded two samples of EO. The extracted two samples of EO were stored in sealed air-tight glass vials at 4 °C until further analysis. The extracted EO constituents were analyzed and recognized by GC–MS analysis. The GC–MS analysis was performed by gas chromatography–mass spectrometry at the department of Medicinal and Aromatic Plants Research, National Research Center, Dokki, Giza, Egypt. This instrument consists of TRACE GC Ultra Gas Chromatographs (THERMO Scientific™ Corporate, Waltham, MA, USA), coupled with a Thermo Scientific ISQ™ EC single quadrupole mass spectrometer. The GC–MS system is equipped with a TR-5 MS column (30 m × 0.32 mm i.d., 0.25 μm film thickness). Helium was used as carrier gas at a flow rate of 1.0 mL min^−1^ with a split ratio of 1:10 following a temperature program of 60 °C for 1 min, rising by 4.0 °C min^−1^ to 240 °C and held for 1 min. Both injector and detector were held at 210 °C. One μL of the diluted samples in hexane at a ratio of 1:10 (*v/v)* was injected. Mass spectra were recorded by electron ionization (EI) at 70 eV, using a spectral range of *m/z* 40–450.

The identification of the chemical constituents of the EOs was achieved using Automated Mass spectral Deconvolution and Identification (AMDIS) software, Wiley spectral library collection, NIST library database, retention indices relative to *n*-alkanes (C_8_-C_22_) or appraisal of the mass spectrum with authentic standards.

### 3.3. Phytotoxic Activity of the Extracted EOs

The phytotoxic activity of the extracted EOs from *T. polium* and *T.decussatus* using HD and MAE methods was assessed on the germination and seedling growth of the test plant *Lactuca sativa* (lettuce). The seeds were purchased from the Agriculture Research Center, Mansoura, Egypt. Seeds with a uniform color and size were selected and sterilized by immersion in a solution of sodium hypochlorite (0.3%) for three min and immediately rinsed three times with distilled and sterilized water [65].

To perform the phytotoxic activity, various concentrations (250, 500, 750, and 1000 µL L^−1^) of *T. polium* and *T. decussatus* EOs were prepared using 1% (*v/v*) Tween^®^80 (Sigma-Aldrich, Darmstadt, Germany) as an emulsifying agent. In sterilized Petri plates (90 mm) lined with filter papers (Whatman No. 1), 20 sterilized seeds were added and 4 mL of each concentration of the EO was poured. The seeds were arranged away from each other, allowing spread within all the plates, and the plate was sealed with Parafilm^®^ tape (Sigma, St. Louis, MO, USA) and kept at 25 °C in a growth chamber with controlled conditions of light (16 h/8 h light/dark cycle). Control plates were prepared as previously mentioned but using Tween 1% alone instead of EO. The plates were checked daily and after six days of incubation, the germinated seeds were counted and the lengths of the root and shoot were measured [65]. The inhibition of seed germination or seedling growth was calculated as follows:

(1) $$\mathrm{Inhibition}\;=100\times \frac{\left(\mathrm{No}/\mathrm{Length}\;\mathrm{of}\;\mathrm{control}-\mathrm{No}/\mathrm{Length}\;\mathrm{of}\;\mathrm{treatment}\right)}{\mathrm{No}/\mathrm{Length}\;\mathrm{of}\;\mathrm{control}}$$

According to the data obtained, all concentrations of the EOs of *T. decussatus* showed complete inhibition, i.e., no germination at all. Therefore, lower concentrations (25, 50, 75, and 100 µL L^−1^) were prepared and the experiment was repeated again.

### 3.4. Antimicrobial Properties of EOs

The extracted EOs of *T. polium* and *T. decussatus* were tested against a panel of pathogenic bacterial and fungal strains using agar diffusion technique, according to Prabuseenivasan, et al. [66] and Rota, et al. [67]. In detail, the EOs were extracted using the HD and MAE methods from the plants under study, *T. polium* and *T. decussatus*, and were tested in vitro against Gram-positive bacteria (*Bacillus subtilis* ATCC6633, *Staphylococcus aureus* ATCC29213), Gram-negative bacteria (*Escherichia coli* ATCC25922), unicellular yeast fungi (*Candida albicans* ATCC10321), and filamentous fungi (*Aspergillus niger* NRC53, *Fusarium solani* NRC15). Bacteria and yeast strains are from the American Type Culture Collection, and fungal isolates were obtained from the culture collection of the Department of Chemistry of Natural and Microbial Products, National Research Center, Cairo, Egypt.

The EOs were individually mounted on sterile paper discs (6 mm in diameter) at a concentration of 10 μL/disc. Thiophenicol and treflucan were used as positive controls for antibacterial and antifungal activities at a concentration of 100 μg/disc. All discs were placed on plates inoculated with 1 × 10^6^ spores^−mL^ of fungi (potato dextrose agar medium) and 1 × 10^8^ spores^−mL^ of bacteria (nutrient agar medium). Inoculated agar plates were left for 30 min at 4 °C for oil diffusion; after that, the plates were incubated for 24 h at 30 °C for bacteria and 72 h at 28 °C for fungi. The diameter of the inhibition zones was measured in millimeters, at three different points, and the average values were reported.

For the determination of the minimum inhibitory concentration (MIC), the plant EOs were tested at the final concentrations, ranging from 1000 to 0.49 µg/mL. The lowest concentration showing an inhibition zone around the disc was taken as the MIC.

### 3.5. Statistical Analysis

The phytotoxic bioassay experiments were performed in a completely randomized design, and the experiment was repeated three times with five replicates per treatment. Based on two factors, the extraction methods (two types) and the concentration (four concentrations), the data was subjected to two-way ANOVA, followed by Duncan’s test at the probability level of 0.05 using the CoStat software program (CoHort Software, Monterey, CA, USA). According to the approach proposed by Legendre and Legendre [68], the data of EO chemical composition extracted by HD and MAE methods were submitted to principal component analysis (PCA), to reproduce a synthetic representation of chemical compounds. PCA was performed using XLSTAT software (version 2018, Addinsoft, NY, USA).

## 4. Conclusions

The present study revealed that MAE method of *T. polium* and *T. decussatus* yielded more EOs than those obtained using HD method, while the number of identified compounds were comparable. The EOs of *T. polium* are characterized by the preponderance of sesquiterpenes, especially 6-epi-shyobunol, delta-cadinene, *α*-pinene, *β*-pinene, germacrene-D, and *t*-muurolol. However, the EOs of *T. decussatus* are dominated by monoterpenes, with carvacrol and *p*-cymene as major compounds. The EOs of *T. decussatus* showed strong phytotoxic, antibacterial, and antifungal activities. However, the *T. polium* EOs revealed moderate phytotoxic and antibacterial activities and no antifungal activity. Therefore, the *T. decussatus* EOs could be integrated as ecofriendly bio-herbicides or as antimicrobial agents. However, further studies are needed for more characterization of the reported major compounds, either singular or in synergism, and to explore their activity at the field scale. On the other hand, from a taxonomic point of view, our results revealed either close similarity or differences of the EO composition of *T. polium* with other ecospecies, therefore we recommend further identification of Egyptian ecospecies of *T. polium*, which could help for more detailed identification at the sublevel.

## Figures and Tables

**Table 1:** Chemical profiles of Essential oils (EOs)of *Teucrium polium* and *Thymus decussatus* extracted using hydrodistillation (HD) and microwave-assisted extraction (MAE) methods.

No.	Compound	Rt	KI_Exp_	KI_Lit_	*T. polium*		*T. decussatus*		Identification ^b^
					HD	MAE	HD	MAE	
**Monoterpene hydrocarbons**									
1	*α*-Thujene	4.57	925	925	0.52 ± 0.01 ^a^	1.26 ± 0.03	0.04 ± 0.01	----	MS, KI
2	*α*-Pinene	4.77	935	936	8.94 ± 0.06	11.58 ± 0.09	----	----	MS, KI
3	Camphene	5.80	954	954	----	0.44 ± 0.02	----	0.30 ± 0.02	MS, KI
4	2,4(10)-Thujadiene	5.96	961	960	----	0.32 ± 0.01	----	----	MS, KI
5	Sabinene	7.44	975	975	2.94 ± 0.04	1.14 ± 0.03	----	----	MS, KI
6	*β*-Pinene	7.54	978	979	7.38 ± 0.05	8.20 ± 0.09	0.09 ± 0.01	0.54 ± 0.03	MS, KI
7	*α*-Myrcene	10.19	986	986	2.90 ± 0.04	----	0.16 ± 0.01	1.04 ± 0.03	MS, KI
8	*p*-Cymene	10.20	1024	1026	----	----	3.61 ± 0.04	16.98 ± 0.10	MS, KI
9	Limonene	10.94	1034	1033	1.10 ± 0.02	0.58 ± 0.03	----	----	MS, KI
10	α-Terpinene	12.18	1018	1018	----	----	----	1.01 ± 0.03	MS, KI
11	β-Terpinene	12.23	1031	1032	----	----	0.23 ± 0.01	----	MS, KI
12	*β*-Phellandrene	12.64	1045	1044	----	----	----	0.21 ± 0.01	MS, KI
13	γ-Terpinene	14.43	1061	1061	0.34 ± 0.02	----	0.49 ± 0.01	2.51 ± 0.06	MS, KI
14	*β*-Terpinolene	15.15	1084	1086	----	----	0.12 ± 0.01	0.34 ± 0.01	MS, KI
**Oxygenated monoterpenes**									
15	*α*-Fenchyl alcohol	12.70	1106	1107	0.39 ± 0.02	----	----	----	MS, KI
16	*cis*-Verbenol	13.65	1142	1144	0.34 ± 0.03	----	----	----	MS, KI
17	Menthol	14.17	1181	1182	----	----	----	0.54 ± 0.01	MS, KI
18	*α*-Terpineol	20.28	1189	1189	0.44 ± 0.03	----	----	----	MS, KI
19	Thymol methyl ether	20.41	1236	1237	----	----	----	0.03 ± 0.01	MS, KI
20	Dihydrocarvone	21.31	1243	1242	----	----	----	0.06 ± 0.01	MS, KI
21	Carvacrol	22.79	1304	1304	----	----	94.40 ± 0.52	75.91 ± 0.61	MS, KI
22	*α*-Terpinenyl acetate	25.21	1349	1350	0.51 ± 0.02	----	----	----	MS, KI
23	Thymyl acetate	28.55	1357	1357	----	----	0.05 ± 0.01	0.06 ± 0.01	MS, KI
**Sesquiterpene hydrocarbons**									
24	*α*-Cubebene	21.37	1351	1351	----	1.10 ± 0.03	----	----	MS, KI
25	*α*-Bourbonene	25.43	1374	1374	----	0.48 ± 0.04	----	----	MS, KI
26	*α*-Copaene	25.77	1377	1376	0.47 ± 0.02	1.34 ± 0.04	----	----	MS, KI
27	*β*-Elemene	26.52	1385	1384	----	0.42 ± 0.03	----	----	MS, KI
28	Longifolene	27.61	1401	1402	----	----	0.07 ± 0.01	----	MS, KI
29	*cis*-Caryophyllene	27.89	1410	1409	0.97 ± 0.03	1.72 ± 0.05	0.33 ± 0.02	0.17 ± 0.01	MS, KI
30	Clovene	29.37	1426	1425	----	----	0.13 ± 0.01	----	MS, KI
31	Aromadendrene	29.55	1440	1439	3.86 ± 0.04	0.33 ± 0.03	0.10 ± 0.01	0.04 ± 0.01	MS, KI
32	*β*-Neoclovene	30.56	1455	1454	----	----	0.04 ± 0.01	----	MS, KI
33	*α*-Gurjunene	30.74	1475	1475	0.68 ± 0.03	----	----	----	MS, KI
34	Germacrene-D	31.99	1480	1480	2.21 ± 0.06	7.25 ± 0.46	----	----	MS, KI
35	*β*-Selinene	33.44	1485	1485	----	----	0.04 ± 0.01	----	MS, KI
36	Guaia-1(10),11-diene	33.72	1488	1488	----	1.69 ± 0.07	----	----	MS, KI
37	*β*-Muurolene	34.08	1493	1493	0.57 ± 0.04	5.55 ± 0.06	----	0.05 ± 0.01	MS, KI
38	*α*-Muurolene	34.54	1498	1499	0.72 ± 0.01	7.64 ± 0.58	----	----	MS, KI
39	δ-Cadinene	35.03	1525	1524	7.04 ± 0.31	25.13 ± 0.43	0.06 ± 0.01	0.08 ± 0.01	MS, KI
40	Cadina-1(10),4-diene	35.89	1530	1530	----	1.83 ± 0.05	----	----	MS, KI
**Oxygenated sesquiterpenes**									
41	Spathulenol	28.61	1515	1516	----	0.51 ± 0.02	----	----	MS, KI
42	1,5-Epoxy-salvial-4(14)-ene	29.64	1548	1548	0.86 ± 0.02	0.78 ± 0.03	----	----	MS, KI
43	Ledol	29.67	1564	1565	0.69 ± 0.04	0.58 ± 0.03	----	----	MS, KI
44	4-epi-Cubebol	29.29	1572	1574	----	0.30 ± 0.01	----	----	MS, KI
45	Germacrene D-4-ol	30.71	1578	1577	5.65 ± 0.31	----	----	----	MS, KI
46	Τ-Cadinol	32.23	1638	1640	2.06 ± 0.05	7.61 ± 0.12	----	----	MS, KI
47	Cubenol	32.31	1641	1642	----	0.35 ± 0.01	----	----	MS, KI
48	Τ-Muurolol	32.74	1646	1646	12.88 ± 0.15	----	----	----	MS, KI
49	*α*-Cadinol	34.16	1654	1653	2.01 ± 0.03	5.49 ± 0.10	----	----	MS, KI
50	Alloaromadendrene oxide	39.89	1672	1672	0.53 ± 0.01	6.09 ± 0.23	----	----	MS, KI
51	6-Epi-shyobunol	41.92	1682	1680	33.00 ± 0.72	0.29 ± 0.01	----	----	MS, KI
**Non-oxygenated hydrocarbons**									
52	Nonadecane	45.04	1900	1900	----	----	0.03 ± 0.01	0.03 ± 0.01	MS, KI
53	Heptacosane	51.54	2700	2700	----	----	0.01 ± 0.00	0.10 ± 0.02	MS, KI
**Monoterpene hydrocarbons**					24.12	23.52	4.74	22.93	
**Oxygenated monoterpenes**					1.68	0	94.45	76.6	
**Sesquiterpene hydrocarbons**					16.52	54.48	0.73	0.34	
**Oxygenated sesquiterpenes**					57.68	22	0	0	
**Non-oxygenated hydrocarbons**					0	0	0.04	0.13	
**Total**					100%	100%	100%	100%	

Rt: retention time, KI_Lit_: published Kovats retention indices; KI_exp_: Kovats index determined experimentally relative to C_8_–C_28_ *n*-alkanes; ^a^ Values are mean ± standard deviation; ^b^ the identification of EO constituents based on the comparison of the mass spectral data and Kovats indices (KI) with those of NIST Mass Spectral Library (2011) and Wiley Registry of Mass Spectral Data 8^th^ edition and literature. HD: hydrodistillation, MAE: microwave-assisted extraction.

**Table 2:** Phytotoxic inhibitory activity of EOs extracted from *Teucrium polium* and *Thymus decussatus* using microwave-assisted extraction (MAE) and hydrodistillation (HD) methods.

Plant Species	Extraction Method	Conc. (µL L^−1^)	Inhibition (%) *		
			Germination	Shoot Growth	Root Growth
*Teucrium polium*	Hydrodistillation	250	19.6 ± 0.95	8.2 ± 0.74	14.9 ± 2.76
		500	33.0 ± 1.50	10.8 ± 0.35	23.2 ± 2.03
		750	43.0 ± 1.98	21.5 ± 1.47	35.8 ± 1.52
		1000	51.9 ± 2.09	31.4 ± 1.63	54.4 ± 1.90
	Microwave-assisted extraction	250	24.1 ± 1.01	0.4 ± 0.35	15.3 ± 3.41
		500	37.5 ± 1.13	6.0 ± 2.03	18.7 ± 2.45
		750	40.0 ± 2.01	17.7 ± 1.44	26.0 ± 2.94
		1000	50.9 ± 2.25	39.8 ± 1.30	42.3 ± 2.75
*p*-value _(0.05)_ Extraction method (E)			0.438 ^ns^	0.0150 *	<0.001 ***
*p*-value _(0.05)_ Concentration (C)			<0.001 ***	<0.001 ***	<0.001 ***
*p*-value _(0.05)_ Interaction (E × C)			0.878 ^ns^	<0.001 ***	0.031 *
*Thymus decussatus*	Hydrodistillation	25	50.9 ± 1.59	33.0 ± 2.37	11.6 ± 0.98
		50	55.4 ± 1.55	45.7 ± 1.42	25.2 ± 3.06
		75	68.8 ± 2.11	52.1 ± 1.48	49.8 ± 1.22
		100	77.7 ± 2.36	85.8 ± 1.18	84.6 ± 1.02
	Microwave-assisted extraction	25	42.0 ± 1.11	45.6 ± 1.18	42.1 ± 2.54
		50	50.9 ± 1.99	58.5 ± 1.78	56.1 ± 1.47
		75	68.8 ± 2.25	65.7 ± 1.18	70.6 ± 1.22
		100	86.6 ± 2.98	87.4 ± 3.55	89.9 ± 2.04
*p*-value _(0.05)_ Extraction method (E)			0.715 ^ns^	<0.001 ***	<0.001 ***
*p*-value _(0.05)_ Concentration (C)			<0.001 ***	<0.001 ***	<0.001 ***
*p*-value _(0.05)_ Interaction (E × C)			0.250 ^ns^	0.007 **	<0.001 ***

* values are means of triplicates ± SE. Different superscript letters within the column of each treatment mean values significant variation at *p* ≤ 0.05 where ^ns^: not significant. The statistical significance was indicated by * *p* ≤ 0.05, ** *p* ≤ 0.01 or *** *p* ≤ 0.001

**Table 3:** Antimicrobial activities of EOs with a concentration of 10 μL/disc against pathogenic bacteria and fungi.

Plant	Extraction Method	Bacteria			Fungi		
		Gram-Positive		Gram-Negative			
		*B. subtilis* ATCC6633	*S. aureus* ATCC29213	*E. coli* ATCC25922	*C. albicans* ATCC10321	*A. niger* NRC53	*F. solani* NRC15
*Teucrium polium*	HD	10.00 ± 0.79 *	10.00 ± 2.12	10.00 ± 0.71	NA	NA	NA
	MAE	11.00 ± 2.12	11.00 ± 0.71	10.00 ± 1.41	NA	NA	NA
*Thymus decussatus*	HD	39.00 ± 0.71	34.00 ± 1.40	35.00 ± 2.12	34.00 ± 2.12	47.00 ± 2.12	40.00 ± 2.83
	MAE	38.00 ± 2.90	35.00 ± 0.70	36.00 ± 1.34	35.00 ± 0.00	48.00 ± 3.14	40.00 ± 1.78
Thiophenicol (100 μg/disc)		30.0 ± 0.71	30.00 ± 0.71	27.00 ± 0.77	25.00 ± 1.55	----	----
Treflucan (100 μg/disc)		----	----	----	29.00 ± 0.70	13.00 ± 0.14	11.00 ± 0.71

* values are the average (*n = 3*) of the inhibition zone diameter (mm) ± standard deviation, HD: Hydrodistillation, MAE: Microwave-assisted extraction. NA: No activity.

**Table 4:** Minimum inhibitory concentrations (MICs) in μg/mL of the EOs extracted using hydrodistillation (HD) and microwave-assisted extraction (MAE) methods against different bacterial and fungal strains.

Plant	Extraction Method	Bacteria			Fungi		
		Gram-Positive		Gram-Negative			
		*B. subtilis* ATCC6633	*S. aureus* ATCC29213	*E. coli* ATCC25922	*C. albicans* ATCC10321	*A. niger* NRC53	*F. solani* NRC15
*Teucrium polium*	HD	62.50	125.00	250.00	----	----	----
	MAE	62.50	125.00	250.00	----	----	----
*Thymus decussatus*	HD	3.91	1.95	0.98	3.91	0.49	7.81
	MAE	3.91	1.95	0.98	3.91	0.49	7.81
Thiophenicol		3.13	3.13	6.25	----	----	----
Treflucan		----	----	----	12.5	50	50
